# Immobilization of Trypsin and Lysozyme in Halloysite Nanotubes for Producing Chitosan Coatings with Antibacterial Properties

**DOI:** 10.3390/polym17233212

**Published:** 2025-12-02

**Authors:** Yuliya Cherednichenko, Ilnur Ishmukhametov, Svetlana Batasheva, Gölnur Fakhrullina, Rawil Fakhrullin

**Affiliations:** 1 Institute of Fundamental Medicine and Biology, Kazan Federal University, 420008 Kazan, Republic of Tatarstan, Russia; 2 Institute for Regenerative Medicine, Sechenov First Moscow State Medical University (Sechenov University), 119991 Moscow, Russia

**Keywords:** halloysite, carboxymethylcellulose, chitosan, nanocomposite, antibacterial coatings, trypsin, lysozyme

## Abstract

A simple method for producing a nanocomposite based on halloysite nanotubes modified with carboxymethylcellulose and trypsin and lysozyme enzymes was developed. Fourier transform infrared spectroscopy confirmed the presence of enzymes in the samples. Chitosan-based coatings were subsequently produced from the nanocomposites. Atomic force microscopy visualization revealed the formation of globular structures consisting of enzymes and carboxymethylcellulose on the halloysite surface. An analysis of the coatings revealed a uniform distribution of halloysite throughout the matrix. The antibacterial activity of the nanocomposite containing lysozyme against *Escherichia coli* OP 50-1 was 47.2% and 63.9% at a concentration of 0.5 and 1 mg/mL, respectively. The antibacterial activity of the nanocomposite containing trypsin against *Escherichia coli* OP 50-1 was 44.4 and 55.5% at concentrations of 0.5 and 1 mg/mL, respectively. The antibacterial activity of the nanocomposite containing lysozyme against *Staphylococcus aureus* 6583 was 26.1 and 50.7% at concentrations of 0.5 and 1 mg/mL, respectively. The antibacterial activity of the nanocomposite containing trypsin against *Staphylococcus aureus* 6583 was 53.6 and 75.4% at concentrations of 0.5 and 1 mg/mL, respectively. The antibacterial activity of coatings based on chitosan and the nanocomposites containing trypsin and lysozyme against *Escherichia coli* OP 50-1 and *Staphylococcus aureus* 6583 was observed.

## 1. Introduction

Nanoarchitectonics, a post-nanotechnology concept aimed at the development of functional materials using nanoscale units, has found applications in antimicrobial materials [1]. A variety of building blocks have been explored, including biological polymers and inorganic materials. Enzyme technologies have crucially changed many industries, ranging from food, textile, and paper industries to medicine and biofuel production. Enzymes act as catalysts for many chemical reactions, binding and acting on their targets with high affinity and specificity [2,3]. As they are soluble and active in aqueous and organic solvents, they find numerous industrial applications [4]. Lysozyme and trypsin, due to their biological activity and low toxicity, as well as antibacterial properties, have attracted considerable attention in food packaging, biomedicine, agriculture, and cosmetics [4,5,6]. However, the requirements for mild temperature and pressure, non-toxic solvents, relatively short period of activity and the specificity of antibacterial action against either Gram-positive or Gram-negative bacteria are the main limitations that have prevented the full implementation of enzymes as an alternative to synthetic antiseptics [7].

To overcome these limitations, point mutations, cross-linking, and immobilization are used [8,9]. Immobilization is a fairly simple method that increases the stability of the enzyme and allows its reuse. Various substrates are applied for immobilization, including zeolites [10], natural and synthetic polymers, and nano/microparticles [11,12]. Some types of clays exert antibacterial activity against bacterial pathogens, including antibiotic-resistant strains, by creating a low pH environment (<4.6) through mineral oxidation, dissolution, and hydrolysis reactions, supporting the release of metals and the production of reactive oxygen species [13].

Halloysite is a particularly interesting substrate due to its size, ranging from 400 to 1000 nm, and its lumen of 15–20 nm, which allows the immobilization of various molecules inside the nanotube, including drugs, antiseptics, anticorrosive agents, and proteins [14,15]. Globular enzymes with a typical diameter of 3–6 nm can be loaded into the nanotube lumen, and then they slowly diffuse outwards. In particular, enzymes were loaded into the lumen of hydroxylated nanotubes, followed by functionalization of the outer surface of h-HNT with a saturated PEI layer [16]. Charged macromolecules can be selectively immobilized on the inner/outer surfaces of halloysite nanotubes [17,18] due to the positive charge of the lumen consisting of aluminum hydroxide, and the negatively charged silica-lined surface. Halloysite nanotubes were used as a carrier for immobilization of laccase, glucose oxidase and lipase [19]. Because of its biocompatibility, halloysite can be included in materials intended for contact with humans and animals [20,21,22]. Thus, the structure, modifiability, and ease of use of halloysite, which does not require energy-intensive purification processes, allow its application in the development of antibacterial coatings.

The widespread availability of chitin and chitosan [23], as well as their low toxicity, high biodegradability, and film-forming properties, have made them ideal materials for immobilization and coating applications [24,25,26]. Sun et al. (2010) showed that halloysite/chitosan (HNTs/Chi) composite films improved the immobilization of horseradish peroxidase (HRP) [27]. In addition, chitosan has been shown to have intrinsic antimicrobial properties due to the presence of free amino groups in its structure [28]. Thus, membranes made of nanotubes and chitosan with immobilized lipase/lysozyme had an antibacterial effect against the bacteria *Micrococcus lysodeikticus* [18]. Films and coatings obtained using chitosan alone are fragile, high permeable, and demonstrate a low bioactive functionality [29]. Doping chitosan with nanoclays is a promising method for the physical modification and improvement of its properties for practical applications [30]. Nanoclays can diffuse in the chitosan matrix and create structures of various nanoparticle arrangements and functionalities [31,32,33].

Despite the studies on the immobilization of enzymes on halloysite, the determination of the antibacterial activity of the obtained nanocomposites has not been sufficiently shown. This study demonstrates the feasibility of modifying halloysite with carboxymethylcellulose, followed by immobilization of trypsin or lysozyme using vacuum infiltration. The resulting nanocontainers were tested for antibacterial activity against *Staphylococcus aureus* 6583 and the antibiotic-resistant *E. coli* OP 50-1. The nanocontainers were incorporated into a chitosan matrix using stirring and ultrasound treatment, which was confirmed by atomic force microscopy.

## 2. Materials and Methods

### 2.1. Materials

Natural biocompatible halloysite clay mineral (HNT) (Sigma-Aldrich, St. Louis, MO, USA), lysozyme from egg white, trypsin from bovine pancreas (SERVA Electrophoresis GmbH, Heidelberg, Germany), carboxymethylcellulose (CMC) (Sigma-Aldrich, Buchs, Switzerland), and medium molecular weight chitosan (with a weight range of 190–310 kDa and a degree of acetylation of 75–85%) (Sigma-Aldrich, St. Louis, MO, USA) were used in this study. Microorganism cultures were grown in liquid LB medium Lennox (Dia-M) and LA agar medium Lennox (Dia-M) with the addition of the antibiotic streptomycin (AppliChem, Darmstadt, Germany) at a concentration of 100 mg/L and Tryptic Soy Agar, Tryptic Soy Broth (BD Bacto™, Franklin Lakes, NJ, USA).

### 2.2. Halloysite Modification

To tailor the halloysite surfaces, CMC at a concentration of 10 mg/mL was dissolved by heating at 90 °C on a magnetic stirrer, and then a HNT suspension at a concentration of 10 mg/mL was added. The specimens were sonicated for 1 min at 51% ultrasonic power (35.7 W), mixed on an orbital shaker for 30 min and washed three times. Coating efficiency was assessed using dynamic light scattering and microelectrophoresis (Zetasizer Nano ZS, Malvern Panalytical Ltd., Malvern, UK) and AFM imaging. The specimens were then dried at 60 °C, ground, and used to prepare nanocomposites containing trypsin and lysozyme.

### 2.3. Preparation of Nanocomposites Based on Halloysite Nanotubes and the Trypsin and Lysozyme Enzymes

To make halloysite–enzyme composites, a suspension of modified HNT was mixed with trypsin or lysozyme in a 1:5 ratio. The specimens were then sonicated for 1 min at 20% power (14 W) in an ice-bath, mixed on a rotator for 30 min, and vacuum infiltrated. The procedure was repeated five times. After enzyme immobilization, the specimens were centrifuged at 6000 rpm for 10 min, and the pellet was washed three times to remove unbound enzymes. Each washing step included addition of Milli Q water to the pellet, thorough pellet resuspending using a vortex mixer, and centrifugation at 6000 rpm for 10 min. Then, the composites were dried at 40 °C and ground. The supernatant was collected immediately after vacuum infiltration and at all washing steps to assess the quantity of immobilized enzymes. The UV–vis absorbance of the supernatants was measured at 280 nm (NanoPhotometer NP-80 Touch, Implen, Munich, Germany), and the quantity of immobilized enzymes was calculated.

The release kinetics of lysozyme and trypsin were studied with UV–vis spectroscopy (NanoPhotometer NP-80 Touch, Implen, Munich, Germany) at 280 nm. An amount of 2 mg of nanocomposites containing lysozyme and trypsin was suspended in 0.2 mL of deionized water with continuous stirring at 40 °C. Samples were collected at 1 h, 4 h, and 24 h, and the volume withdrawn was replenished with a fresh portion of water.

### 2.4. Determining the Nanotube Modification with CMC and Enzymes

Enzyme immobilization was studied by dynamic light scattering and electrophoretic light scattering using a Zetasizer Nano ZS instrument and standard U-shaped cuvettes (Malvern Panalytical Ltd., Malvern, UK). ATR–Fourier transform infrared spectroscopy (FT-801, Simex, Novosibirsk, Russia) with ZnSe and Ge ATR crystals was also used to study the enzyme immobilization. The spectra were collected in the wavenumber range of 520–4000 cm^−1^ with a resolution of 4 cm^−1^, averaged over 26 scans, baseline corrected and normalized.

### 2.5. Hyperspectral and Dark-Field Microscopy of Halloysite Nanocomposites

Data on halloysite in the nanocomposites were then collected using an Olympus BX51 upright microscope (Olympus, Tokyo, Japan) equipped with a CytoViva^®^ darkfield condenser with a Fiber-Lite DC-950 halogen light source (150 W) (Dolan Jenner Industries Inc., Boxborough, MA, USA) and a ProScan III control module (JH Technologies, Fremont, CA, USA), as described elsewhere [34]. Images were acquired using Exponent 7 imaging software (Dage-MTI, Michigan City, IN, USA). Spectra were recorded using an ImSpector V10E spectrometer (Specim, Oulu, Finland) and a CCD camera. For darkfield microscopy, exposure and signal gain parameters were varied to achieve optimal contrast, while hyperspectral imaging was performed at a fixed exposure time of 0.25 s to ensure quantitative data comparability. The resulting hyperspectral cubes had dimensions of 696 × 675 × 478 (X × Y × λ), where the spatial dimensions corresponded to the microscope field of view, and the spectral dimension included 478 channels in the 400–1000 nm range. Hyperspectral data were recorded using ENVI version 4.8 software (Harris Geospatial Solutions, Broomfield, CO, USA). Raw spectral data were used in this study, since correcting the hyperspectral data for the illumination source significantly reduced the mapping efficiency. Primary segmentation of regions of interest was performed using the maximum likelihood method to separate objects from the background. The identified regions of interest were used to form a spectral library of halloysite, where each reference spectrum represented the average spectrum of a particle. To identify halloysite, a spectral angle mapping algorithm was used, previously tested on reference halloysite images. The final classification of halloysite in complexes was carried out with threshold values of spectral angle up to 0.2 radians.

### 2.6. Atomic Force Microscopy

To visualize the surface topography of halloysite before and after enzyme immobilization, and of the coatings based on chitosan and the nanocomposites, atomic force microscopy was performed using a Dimension Icon microscope (Bruker, USA), operating in PeakForce Tapping mode in air, at room temperature and humidity. ScanAsyst-Air triangular silicon nitride probes (Bruker, USA) with a tip radius of 2 nm, a nominal cantilever length of 115 μm, and a spring constant of 0.4 nm^−1^ were used. Halloysite specimens were suspended in distilled water at a concentration of 0.01 mg/mL, sonicated, and then applied to the surface of freshly cleaved mica. Mica was pre-fixed to the surface of metal disks for atomic force microscopy. To study the films, specimens containing 0.1, 0.5, and 1 mg/mL of nanocomposite in a chitosan solution were applied to glass slides and dried at room temperature. Scanning was performed in PeakForce Tapping QNM mode. Scans were obtained at a resolution of 512 points per line, at a frequency of 0.977 Hz; the peak force was determined experimentally in each case. The size and aspect ratio of the scans were determined based on the structure of the specimen being imaged. The scan data was processed and analyzed using Nanoscope Analysis 3.0 (Bruker, USA) software. During processing, the obtained data was leveled (zero- or first-order surface leveling), defective bands were removed if necessary, and particle sizes and surface roughness characteristics were determined using the software’s algorithms. Statistical analysis was performed using one-way ANOVA with Tukey’s post hoc test.

### 2.7. Antibacterial Activity of Halloysite Nanocomposites

*S. aureus* 6583 (ATCC^®^, Manassas, VA, USA) and Streptomycin-resistant *E. coli* OP 50-1 strain (Caenorhabditis Genetics Centre, Minneapolis, MN, USA) were used as test cultures. Microorganism cultures were grown at 37 °C for 18 h. A bacterial suspension at a concentration of 0.1 OD at a wavelength of 595 nm in a nutrient medium was then incubated with nanocontainers at concentrations of 0.5 and 1 mg/mL for 24 h. Native halloysite tubes at the same concentrations, as well as an untreated *E. coli* OP 50-1 or *S. aureus* 6583 culture, were used as controls. The antibacterial activity of halloysite, containing lysozyme and trypsin, against *E. coli* OP 50-1 or *S. aureus* 6583 was determined by plating bacteria mixed with the enzyme-carrying nanotubes on agar nutrient media and counting viable cells based on the number of colony-forming units (CFUs) per unit volume of suspension.

### 2.8. Preparation of Coatings Based on Halloysite Nanocomposites and Chitosan

To obtain the coatings, a 1% chitosan solution in 1% acetic acid and nanocomposites loaded with either trypsin or lysozyme at ratios of 1, 5, and 10% were used. The coating solutions were sonicated for 5 s at 20% power (14 W) to achieve a more uniform mixing. Then, 0.1 mL of each coating solution was applied to a glass or plate and dried at room temperature.

### 2.9. Determination of the Antibacterial Activity of the Coatings

The antimicrobial activity of the coatings against *E. coli* OP 50-1 or *S. aureus* 6583 was determined by plotting a growth curve. Cultivation was carried out in a 96-well plate with continuous, vigorous shaking and hourly optical density measurements for 24 h using a Multiscan FC microplate photometer (Thermo Scientific, Waltham, MA, USA). The bacterial culture was added to the test wells at a concentration of 0.1 OD at a wavelength of 595 nm in a nutrient medium. The nanocomposite content in the coatings was 1 mg/mL. The growth curve was plotted and the data were statistically processed using GraphPad Prism 10.1.1 (GraphPad Software, Inc., Boston, MA, USA). To provide statistical analysis of the antibacterial action of the coatings, the optical densities after 2 h of cultivation were compared using a two-tailed Student’s t-test with Bonferroni correction.

### 2.10. Statistical Analysis

Data are presented as mean ± standard deviation. All measurements were performed with a minimum of three independent replicates. The exact number of replicates is shown in table captions and figure legends. Surface roughness parameters were analyzed using one-way analysis of variance (ANOVA) with Tukey’s post hoc test for multiple comparisons. Data organization and statistical analysis were performed using GraphPad Prism version 10.1.1 (GraphPad Software, Inc., Boston, MA, USA). Hydrodynamic diameters and zeta-potential values were compared using a two-sided Student’s *t*-test with Bonferroni correction. Groups were considered significantly different at *p* < 0.05.

## 3. Results and Discussion

### 3.1. Composite Structure Investigation

#### 3.1.1. Hydrodynamic Diameter and Zeta Potential of Nanocomposites

Cellulose itself does not have antimicrobial properties, but studies show that it is a suitable carrier for lysozyme in various applications [35]. The interaction of cellulose with lysozyme and other enzymes can stabilize the composite and thereby prolong the antimicrobial activity [36,37]. In addition, it offers such advantages as availability, renewability, ease of modification, biocompatibility, and biodegradability [38]. The HNT modification with CMC (an anionic polyelectrolyte) was demonstrated by a change in the zeta potential of HNT from −27.6 ± 0.3 to −35.5 ± 0.1 mV. The hydrodynamic diameter of nanotubes increased after the modification with CMC as compared to pristine nanotubes. We suggest that a size increase in HNT_CMC compared to pristine HNT was brought about by CMC immobilization on the positive sites located on the halloysite outer surface and nanotube ends. As we have recently demonstrated [39], the negatively charged outer surface of halloysite bears some positive charges at surface defect sites, which support the tight binding of negatively charged polystyrene particles. Studies show that the adsorption of charged polymers on the halloysite surface leads to a shift in the ζ-potential values [40,41].

The HNT_CMC composite was further used for enzyme immobilization using the vacuum infiltration technique. According to UV–vis measurements, 0.19 ± 0.07 mg of lysozyme and 0.22 ± 0.07 mg of trypsin were immobilized per 1 mg of HNT-CMC.

It was found that after the immobilization of trypsin and lysozyme enzymes on HNT, the zeta potential shifted to −18.8 ± 0.7 and −24.5 ± 0.8 mV, respectively, with a small but statistically significant increase (*p* = 4.18 × 10^−5^ for trypsin and *p* = 0.003 for lysozyme) in the hydrodynamic diameter (Table 1). The higher hydrodynamic diameter of the HNT_trypsin composite compared to that of the HNT-lysozyme composite could be related to the different sizes of the enzymes immobilized on the nanotube surface. Trypsin from bovine pancreas (223 amino acid residues, MW 23.8 kDa) is about two times as large as lysozyme from egg white (147 amino acid residues, MW 14.3 kDa).

#### 3.1.2. Fourier Transform Infrared Spectroscopy of Nanocomposites

**Figure 1:**
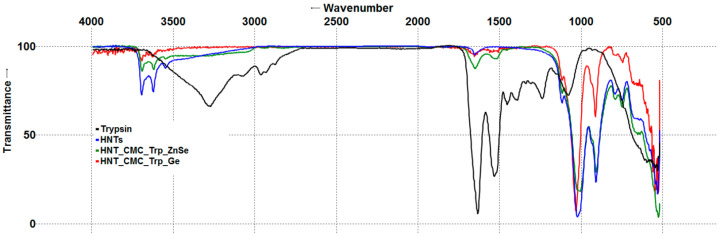
Fourier transform infrared spectra of halloysite composites with trypsin (trypsin and halloysite spectra were obtained using the ZnSe crystal).

**Figure 2:**
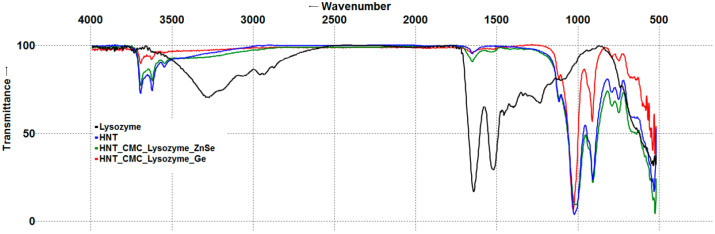
Fourier transform infrared spectra of halloysite composites with lysozyme (lysozyme and halloysite spectra were obtained using the ZnSe crystal).

The infrared spectra of all halloysite-containing specimens were dominated by bands ascribed to two Al_2_OH bending vibrations (940 and 915 cm^−1^) and Si–O–Si stretching vibrations (about 1030 cm^−1^), characteristic of clay minerals. Only slight differences were observed between FTIR spectra of pristine HNT and HNT_CMC, which included a broad blob corresponding to O-H stretching vibrations above 3000 cm^−1^, C-O stretches at about 1200 cm^−1^, and two very small peaks at 1420 and 1455 cm^−1^ appearing in the HNT_CMC spectrum (Supplementary Figure S1). After enzyme immobilization, an infrared spectral analysis revealed the presence of amide group vibrations in the range of 1700–1600 and 1600–1500 cm^−1^ (so-called Amide I and Amide II bands, respectively), characteristic of lysozyme and trypsin, in all nanocomposites studied (Figure 1 and Figure 2) [42,43,44]. Thus, the FTIR analysis confirmed the presence of enzymes in the nanocomposites. One cannot exclude the possibility that some enzyme molecules could be immobilized in the nanotube lumen, considering the negative charge of the lumen and the positive charge of the enzyme molecules and the use of vacuum infiltration technique for enzyme immobilization. While recognizing the very limited applicability of ATR-FTIR for depth profiling [45], we nonetheless attempted to use this method to study the presence of enzymes within the nanotube lumen. For this reason, two ATR crystals, Ge and ZnSe, were used. Ge has a higher refractive index than ZnSe and, therefore, a smaller evanescent wave penetration depth. Because of this property, Ge ATR crystals are sometimes used to maximize the signal from the surface and minimize the signal from the bulk material [45] or solution [46].

Characteristic polypeptide amide bands were observed in composite spectra when using both Ge and ZnSe ATR crystals. Because of deeper penetration of evanescent wave into the ZnSe crystal, spectra collected using the ZnSe crystal were more intense than those collected using the Ge crystal. After all spectra were normalized to 100%, in the nanocomposite spectra obtained on the Ge crystal, the most prominent band associated with external silica layer (about 1030 cm^−1^) was almost equal to or higher in intensity than the same band in the spectra obtained on the ZnSe crystal. However, the bands associated with inner alumina layer (940 and 915 cm^−1^) were weaker on the Ge crystal compared to those on the ZnSe crystal. If enzyme molecules had been collocated with Si-O on the external halloysite surface, one could also expect high intensity of amide bands (about 1640 and 1518 cm^−1^) on the Ge crystal. However, a decrease in amide bands intensities relative to the Si-O band intensity was observed when using the Ge crystal, similarly to the decrease in Al_2_OH bands intensities. Thus, we assume that some enzyme molecules could be located inside the nanotubes.

The assay of the trypsin release kinetics from its nanocomposite with HNT-CMC revealed an initial burst release in the first 4 h due to surface desorption, with a continuous release profile for the next 20 h of observation (Supplementary Figure S2). Lysozyme was not released from its nanocomposite with HNT-CMC during 24 h of the release kinetics study. We suggest that the strong interaction of lysozyme with CMC probably prevented its release from the nanocomposite, but the immobilized lysozyme retained its antibacterial effect. It was previously observed that various enzymes immobilized in halloysite nanotubes retained their enzymatic activity and exhibited enhanced stability under harsh conditions [19,28].

#### 3.1.3. Composite Hyperspectral Analysis and Dark-Field Microscopy

**Figure 3:**
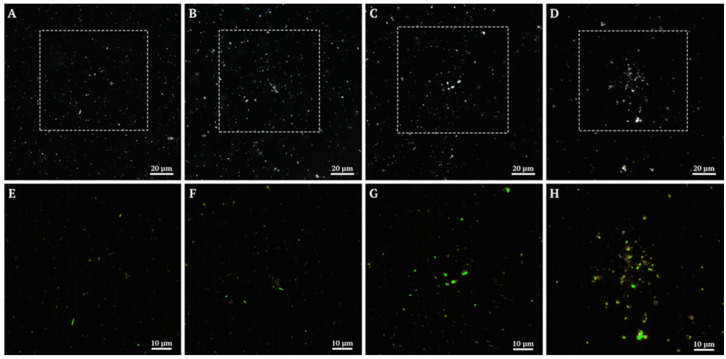
Dark-field microscopy and spectral mapping of halloysite in complexes: (**A**,**E**) halloysite, (**B**,**F**) halloysite-carboxymethylcellulose, (**C**,**G**) halloysite-carboxymethylcellulose-lysozyme, and (**D**,**H**) halloysite-carboxymethylcellulose-trypsin. (**A**–**D**) Dark-field micrographs of the specimens. (**E**–**H**) Spectral mapping results in the areas marked with dashed squares. Identified halloysite is marked in green.

A comprehensive analysis of the specimens using dark-field microscopy and hyperspectral imaging allowed characterization of both the morphological and spectral properties of the studied materials (Figure 3, Supplementary Figure S3). Dark-field images demonstrate certain differences in the morphology and distribution of particles depending on the specimen composition, ranging from dispersed structures in control specimens to the formation of larger aggregates in multicomponent systems (Figure 3A–D). Hyperspectral mapping of the areas highlighted by dashed squares successfully identified the inorganic component in the studied complexes. In the control halloysite specimen, the identified signals are uniformly distributed (Figure 3D). In specimens containing CMC and protein components, areas with concentrated halloysite signals are present, which may indicate material aggregation in the presence of polymer molecules (Figure 3F–H).

#### 3.1.4. Atomic Force Microscopy of Halloysite-Based Composites

**Figure 4:**
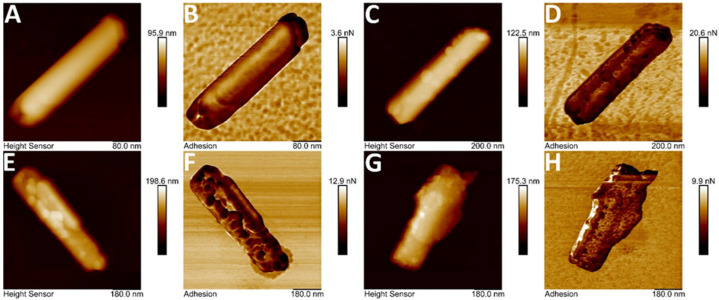
Atomic force microscopy images (topography and non-specific adhesion). (**A**,**B**)—unmodified halloysite; (**C**,**D**)—halloysite modified with carboxymethylcellulose; (**E**,**F**)—with carboxymethylcellulose and lysozyme; (**G**,**H**)—with carboxymethylcellulose and trypsin.

The visualization of halloysite nanotubes using atomic force microscopy allowed identification of trypsin and lysozyme attachment sites on the outer surface of the mineral. Images of isolated nanotubes were obtained in topography and nonspecific adhesion modes (Figure 4). AFM imaging confirmed carboxymethylcellulose immobilization on the silica surface (Figure 4C,D), likely at sites where structural defects occur [39]. Subsequently, enzymes immobilized on CMC binding sites, forming globular structures with a diameter of 50–100 nm. This significantly increases the observed surface unevenness of the nanotubes and increases their surface roughness. The results confirmed enzyme immobilization on the halloysite surface.

### 3.2. Antibacterial Activity of Nanocomposites

**Figure 5:**
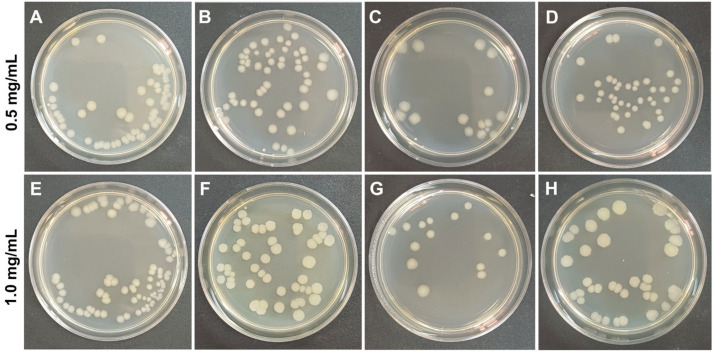
Concentration of *E. coli* OP 50-1 after 24 h cultivation with nanocomposites containing lysozyme and trypsin at a nanocomposite concentration of 0.5 mg/mL and 1 mg/mL: (**A**,**E**)—culture without nanocomposites; (**B**,**F**)—culture with unmodified halloysite; (**C**,**G**)—culture with halloysite modified with carboxymethylcellulose and lysozyme; (**D**,**H**)—culture with halloysite modified with carboxymethylcellulose and trypsin. The diameter of the petri dish is 9 cm.

**Figure 6:**
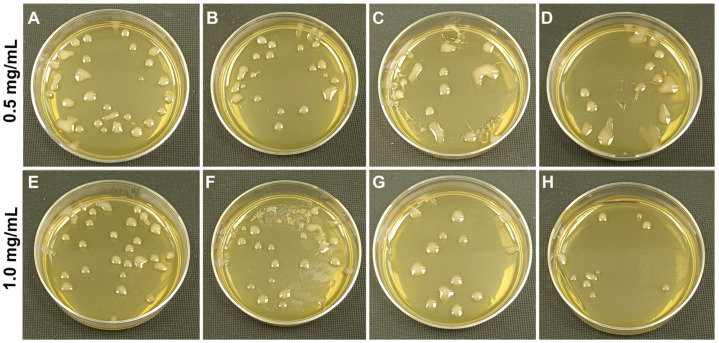
Concentration of *S. aureus* 6583 after 24 h cultivation with nanocomposites containing lysozyme and trypsin at a nanocomposite concentration of 0.5 mg/mL and 1 mg/mL: (**A**,**E**)—culture without nanocomposites; (**B**,**F**)—culture with unmodified halloysite; (**C**,**G**)—culture with halloysite modified with carboxymethylcellulose and lysozyme; (**D**,**H**)—culture with halloysite modified with carboxymethylcellulose and trypsin. The diameter of the petri dish is 9 cm.

**Figure 7:**
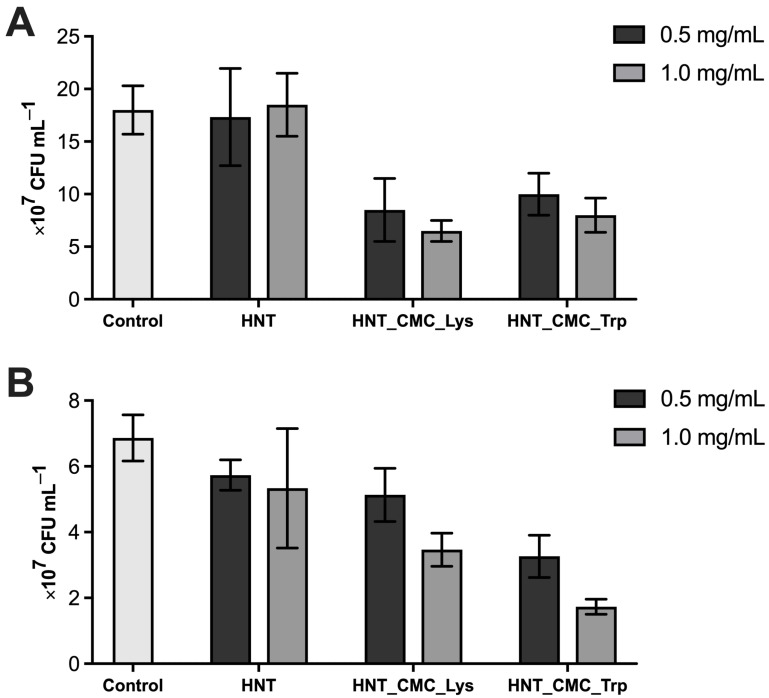
The number of viable cells of *E. coli* OP 50-1 (**A**) and *S. aureus* 6583 (**B**) after 24 h of cultivation with nanocomposites containing lysozyme and trypsin at concentrations of 0.5 and 1 mg/mL (*n* = 3).

Testing of antibacterial activity is the only way to assess lysozyme enzymatic activity [47]. Ultrasound treatment at appropriate frequencies and intensity levels is often applied for enzyme immobilization and it can result in even higher enzyme activity [48]. Studies of the antibacterial activity of the nanocomposites revealed that specimens containing lysozyme were more efficient against *E. coli* OP 50-1, while the specimens containing trypsin were efficient against *S. aureus* 6583 (Figure 5, Figure 6 and Figure 7).

Thus, the antibacterial activity of lysozyme-containing nanocomposites reduced the viable cell count of the streptomycin-resistant *E. coli* strain OP 50-1 by a factor of 2 at a concentration of 0.5 mg/mL and by a factor of 3 at 1 mg/mL. In a specimen containing trypsin, the concentration of viable cells depended on the number of nanocomposites added. The higher the concentration of trypsin-containing nanocomposites, the higher the antibacterial activity was. A similar study by Y. Wang (2015) [49] also demonstrated that lysozyme immobilized on a halloysite composite with layered double hydroxide (anionic clay) exhibited more effective antibacterial activity against *E. coli* than free lysozyme. When studying the antibacterial activity of the nanocomposite against *S. aureus* 6583, the best antibacterial effect was observed for the nanocomposite with trypsin: at a concentration of 0.5 mg/mL, the number of viable cells decreased by a factor of 2, at 1 mg/mL by a factor of 3. Whereas in samples with lysozyme, the antibacterial effect for *S. aureus* was lower than that for *E. coli* OP 50-1. It was shown that lysozyme in nanocomposites exhibited lower antimicrobial activity against *S. aureus* [50]. In samples with native halloysite nanotubes, a slight decrease in viable cells of *S. aureus* 6583 was also observed, which may be associated with the ability of halloysite to exert an antibacterial effect [13].

### 3.3. Atomic Force Microscopy of the Coatings

**Figure 8:**
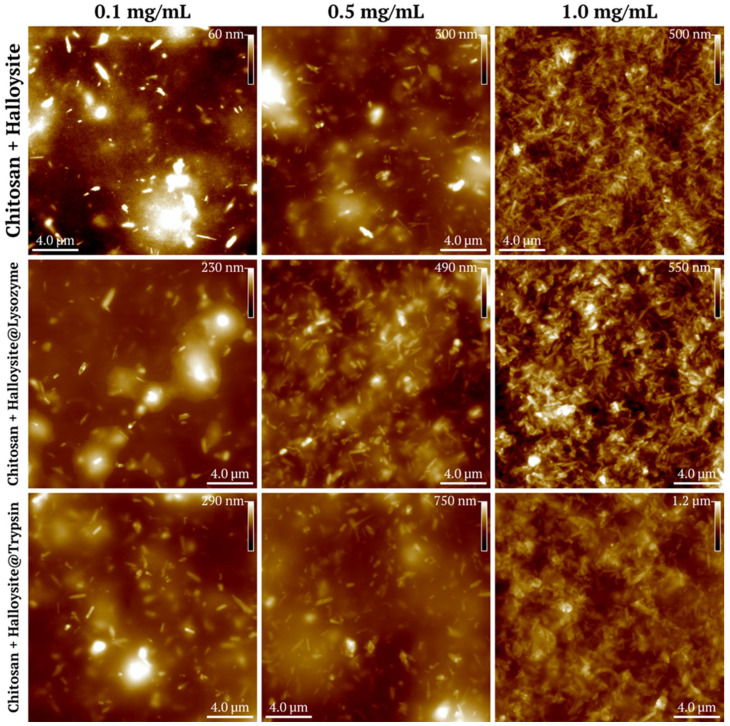
Atomic force microscopy images (topography) of chitosan-based nanocomposite films at varying halloysite concentrations. Representative AFM topography images (20 × 20 μm scan area) of chitosan films incorporating halloysite nanotubes (top row), halloysite@lysozyme complexes (middle row), and halloysite@trypsin complexes (bottom row) at concentrations of 0.1, 0.5, and 1.0 mg/mL. Height scale bars indicate maximum peak heights for each sample.

The nanocomposites were then added to an aqueous chitosan solution at various concentrations to form coatings on the glass surface. Chitosan films were prepared with halloysite at concentrations of 0.1, 0.5, and 1.0 mg/mL, with and without lysozyme or trypsin additives (Figure 8).

Next, we conducted studies of the surface roughness parameters of chitosan films with halloysite nanotubes with immobilized enzymes. The parameters measured include average roughness (Sa), maximum peak height (Sp), root mean square roughness (Sq), maximum valley depth (Sv), and maximum valley-to-peak height (Sz). Data are presented as mean ± standard deviation in nanometers.

**Figure 9:**
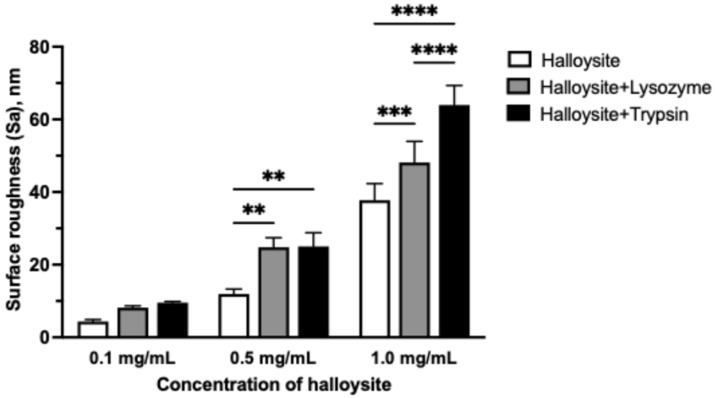
Concentration-dependent effects of halloysite nanotubes and enzyme additives on surface roughness of chitosan films. Data are presented as mean ± standard deviation (*n* = 3–9 per group). Statistical analysis was performed using one-way ANOVA with Tukey’s post hoc multiple comparisons test. Horizontal brackets indicate significant differences between the treatment groups within each concentration (** *p* < 0.01, *** *p* < 0.001, **** *p* < 0.0001).

An AFM analysis of chitosan films showed a baseline average surface roughness value of 0.96 ± 0.09 nm. The addition of halloysite at a concentration of 0.1 mg/mL produced no significant changes compared to the control samples (Table 2). However, significant surface modification occurred when the concentration was raised to 0.5 mg/mL, with the average roughness reaching 11.96 ± 1.30 nm, while a 1.0 mg/mL concentration further elevated roughness to 37.75 ± 4.59 nm. The samples containing lysozyme and trypsin demonstrated similar concentration-dependent behavior, showing no significant differences from controls at 0.1 mg/mL but noticeable surface modification at higher halloysite concentrations. Moreover, both enzymes amplified the surface enhancement effect beyond that produced by halloysite alone. Trypsin demonstrated the strongest influence on surface modification, resulting in maximum roughness values of 64.03 ± 5.36 nm at 1.0 mg/mL of halloysite. These results indicate that halloysite incorporation follows concentration-dependent patterns, while the enzymatic functionalization of halloysite provides additional surface enhancement (Figure 9).

It is known that biofilm formation is initiated by the adhesion of microorganisms to the substrate. This process is affected by various parameters on both the microorganism (duration of exposure, number of bacteria, and specific bacterial properties) and the substrate part (surface charge density, wettability, roughness, stiffness, and topography) [51]. Particularly, surface topography alone can significantly alter bacterial growth in a non-monotonic way. For instance, in a study where quartz slides were selectively etched to obtain hydrophobic surfaces with root mean square roughness ranging from 1.7 to 385 nm, the optimal value for adhesion of *Salmonella*, *Listeria*, and *E. coli* was found to be between 10 and 40 nm, while coatings with roughness outside this range exhibited significantly reduced bacterial attachment [52].

In our work, we observed an increase in root mean square roughness from approximately 1 nm in chitosan-only coatings to 81 nm in chitosan with embedded halloysite@trypsin particles at 1 mg/mL, whereas the coatings with lysozyme showed values of approximately 61 nm. The consistently higher roughness values for trypsin-containing coatings can be attributed to the enzyme’s larger size and structural characteristics. Trypsin (MW ~24 kDa), being a larger enzyme than lysozyme (MW ~14.3 kDa), produces more pronounced topographical modifications when incorporated into halloysite particles. Notably, de Morais et al. also observed that trypsin generated higher surface roughness than lysozyme, although in their study the enzymes were deposited directly on substrates [53].

**Figure 10:**
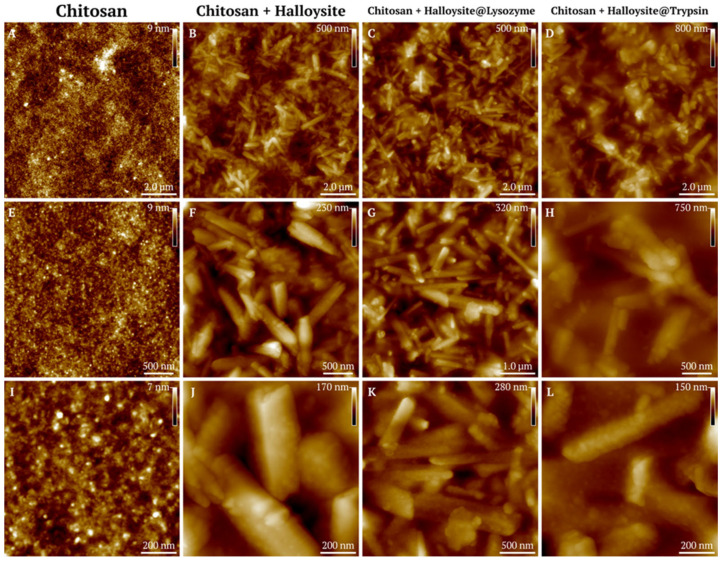
AFM characterization of chitosan-based nanocomposite films at a halloysite concentration of 1.0 mg/mL. Representative AFM topography images of pristine chitosan film (**A**,**E**,**I**) and chitosan films incorporating halloysite nanotubes (**B**,**F**,**J**), halloysite@lysozyme complexes (**C**,**G**,**K**), and halloysite@trypsin complexes (**D**,**H**,**L**).

**Figure 11:**
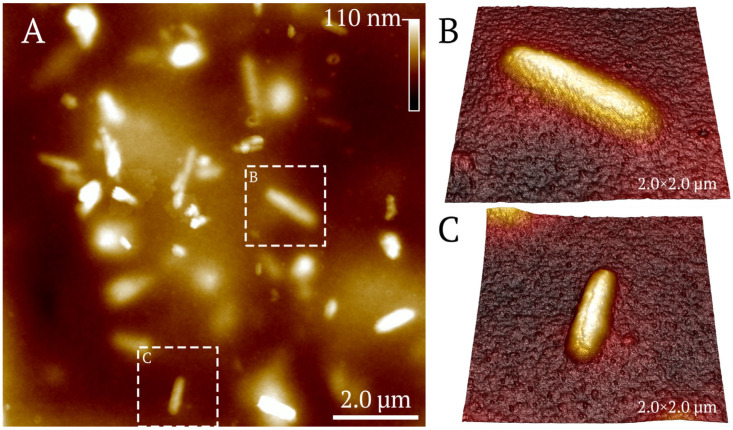
AFM topography and three-dimensional reconstruction of chitosan + halloysite@trypsin films at a concentration of halloysite of 0.1 mg/mL. Two-dimensional topography image (**A**) with isolated halloysite nanotubes dispersed across the chitosan film surface. Dashed boxes (**B**,**C**) indicate regions with individual halloysites shown in three-dimensional perspective views.

The surface topography studied with AFM (Figure 10) demonstrated nanocomposites embedded in the chitosan matrix, with the nanotubes randomly located in the coatings after drying. In addition, in Figure 11B,C, the chitosan coating on the halloysite surface is clearly visible.

### 3.4. Antibacterial Activity of the Coatings

**Figure 12:**
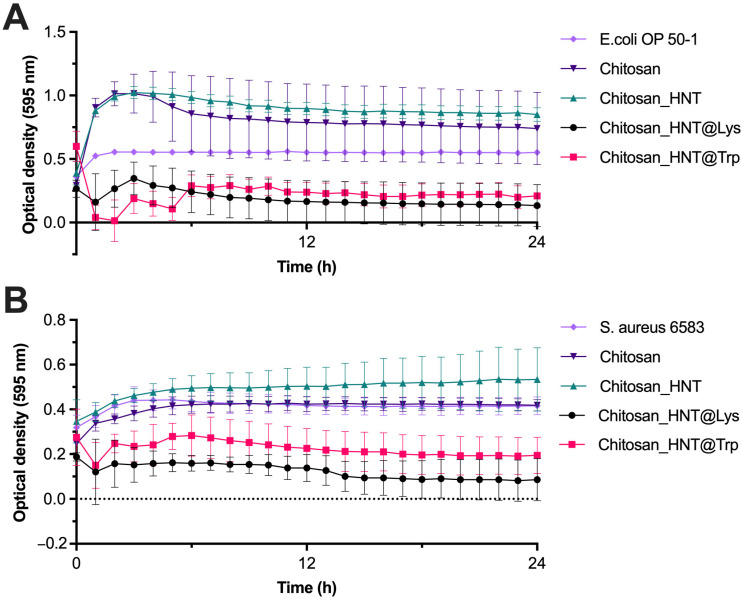
Growth curve of *E. coli* OP 50-1 (**A**) and *S. aureus* 6583 (**B**), demonstrating an antibacterial effect of the coatings at a concentration of 1 mg/mL (*n* = 3–6).

The antibacterial activity of the coatings against *E. coli* OP 50-1 and *S. aureus* 6583 was studied using the spectrophotometric method and subsequent plotting of growth curves (Figure 12).

The chitosan-based coatings with nanocomposites exhibited antibacterial activity against the Gram-positive *S. aureus* strain 6583 and the Gram-negative *E. coli* OP 50-1. The 24-h analysis ensures a high cell concentration in the sample, allowing for accurate counting and assessment of viable microorganisms. The antibacterial effect was observed 1 h after bacteria culturing on the coatings and persisted for 24 h of this study. To provide the statistical analysis of the antibacterial action of the coatings, the optical densities after 2 h of cultivation were compared using Student’s t-test with Bonferroni correction. Both coatings exerted an antibacterial effect against *E. coli* (with a *p*-value of 5.39 × 10^−6^ for Chitosan_HNT_Lys and a *p*-value of 1.77 × 10^−6^ for Chitosan_HNT_Trp, as compared to the bacterial culture control) and *S. aureus* (with a *p*-value of 0.003 for Chitosan_HNT_Lys and a *p*-value of 0.003 for Chitosan_HNT_Trp, as compared to the bacterial culture control). The most pronounced antibacterial effect was observed after 2 h and lasted for 4 h when culturing *E. coli* OP 50-1 on coatings with Chitosan_HNT_Trp. When culturing *S. aureus* 6583, a more pronounced antibacterial effect was observed on the Chitosan_HNT_Lys coatings. However, in all studies, statistically significant differences between the Chitosan_HNT_Trp and Chitosan_HNT_Lys coatings were not observed (with a *p*-value of 0.10 in the *E. coli* study and a *p*-value of 0.24 in the *S. aureus* study). Furthermore, chitosan and Chitosan-HNT-based coatings were found to stimulate growth when cultured with *E. coli* OP 50-1. Liu N. et al. found that chitosan had a stimulating effect on the growth of *E. coli*, depending on its concentration and molecular weight [54]. Similar to our study, some studies found no effects when culturing *S. aureus* with chitosan [55,56].

## 4. Conclusions

Modification of halloysite with carboxymethylcellulose ensured the attachment and retention of the lysozyme and trypsin enzymes on the nanotube surface by forming a polymer complex, which was confirmed by atomic force microscopy. Sites of carboxymethylcellulose attachment with subsequent enzyme immobilization were identified. The polymer complex remained on the surface even after three washings of specimens. The presence of enzymes was confirmed by IR spectroscopy.

Due to the growing number of antibiotic-resistant strains, the streptomycin-resistant *E. coli* OP 50-1 strain was chosen as a test culture for the antibacterial activity of nanocomposites. In addition, the antibacterial activity against the Gram-positive strain *S. aureus* 6583 was studied. The nanocontainers with lysozyme exhibited more pronounced antibacterial activity. At concentrations of 0.5 and 1 mg/mL, the antibacterial efficacy was 55.6% and 62.9%, respectively. Lower antibacterial activity was observed for nanocontainers with trypsin, which was highly dependent on their concentration in the medium. Thus, at a nanocontainer concentration of 0.5 mg/mL, the antibacterial effect was 27.8%, and at 1 mg/mL—55.6%. This may be due to the lower amount of enzyme in this sample, since trypsin does not form a complex with carboxymethylcellulose as effectively as lysozyme. It was established that the nanocomposite with lysozyme was more efficient against Gram-negative *E. coli* OP 50-1, and the nanocomposite with trypsin was more active against Gram-positive *S. aureus* 6583. Although higher efficiency of lysozyme against Gram-positive bacteria compared to Gram-negative ones is usually reported, in our study, the complexation with carboxymethylcellulose and immobilization on halloysite presumably enhanced the activity of lysozyme against Gram-negative microorganisms. Additionally, the degree of lysozyme resistance can vary in different isolates.

Further study of nanocomposite-based coatings demonstrated the antibacterial activity of coatings containing lysozyme and trypsin. Our results are consistent with those of other studies demonstrating higher stability and antibacterial activity of enzymes in complexes with nanoclay, chitosan, and carboxymethylcellulose [57,58,59]. This paper presents an effective method for immobilizing enzymes on halloysite and creating a stable enzyme-polyelectrolyte complex. Both enzyme-containing samples exhibited antibacterial activity against both Gram-positive and Gram-negative bacteria, making them promising for use in antibacterial coatings.

## Figures and Tables

**Table 1:** Hydrodynamic diameter and zeta potential of pristine and CMC-modified halloysite and nanocomposites based on lysozyme and trypsin (*n* = 3). All values differed significantly from those in the control (pristine HNT) at *p* < 0.017 (Bonferroni-adjusted *p*-value).

Nanoparticle	Hydrodynamic Diameter, nm	Zeta Potential, mV
HNT	284.5 ± 5.3	−27.6 ± 0.3
HNT + CMC	369.7 ± 10.7	−35.5 ± 0.1
HNT + CMC + lysozyme	460.7 ± 7.7	−24.5 ± 0.8
HNT + CMC + trypsin	501.6 ± 22.4	−18.8 ± 0.7

**Table 2:** Surface roughness parameters of chitosan films with halloysite nanotubes and enzyme additives. Data are presented as mean ± standard deviation (*n* = 3–9 per group). Surface parameters were obtained from 10 × 10 µm AFM scans. Statistical analysis was performed using one-way ANOVA with Tukey’s post hoc test.

Sample		Sa, nm	Sp, nm	Sq, nm	Sv, nm	Sz, nm
Chitosan		0.96 ± 0.09	15.76 ± 4.01	1.26 ± 0.12	−4.85 ± 0.45	20.62 ± 3.95
Chitosan + Halloysite, mg/mL	0.1	4.35 ± 0.54	94.35 ± 13.30	7.47 ± 1.13	−15.88 ± 4.24	110.6 ± 14.50
	0.5	11.96 ± 1.30 *	123.7 ± 33.55 *	17.30 ± 2.11 *	−59.04 ± 10.39 *	182.6 ± 31.88
	1.0	37.75 ± 4.59 *	169.3 ± 64.10 *	47.57 ± 5.78 *	−149.3 ± 24.09 *	352.2 ± 52.86 *
Chitosan + Halloysite + Lysozyme, mg/mL	0.1	8.16 ± 0.51	104.3 ± 42.71	11.87 ± 0.93	−25.97 ± 4.50	130.4 ± 39.91
	0.5	24.83 ± 2.60 *	166.0 ± 22.61 *	32.43 ± 3.21 *	−106.9 ± 18.48 *	273.0 ± 13.11 *
	1.0	48.16 ± 5.80 *	293.0 ± 78.34 *	61.03 ± 7.90 *	−200.3 ± 35.93 *	493.4 ± 100.4 *
Chitosan + Halloysite + Trypsin, mg/mL	0.1	9.54 ± 0.30	105.9 ± 7.09	13.88 ± 0.43 *	−46.08 ± 14.10	151.8 ± 11.87
	0.5	25.03 ± 3.78 *	189.0 ± 48.51 *	32.87 ± 4.79 *	−106.0 ± 24.05 *	295.0 ± 63.69 *
	1.0	64.03 ± 5.36 *	349.4 ± 57.87 *	80.99 ± 8.16 *	−249.8 ± 49.03 *	589.0 ± 73.37 *

* Indicates statistically significant differences compared to the chitosan control (*p* < 0.05).

## Data Availability

The original contributions presented in the study are included in the article/Supplementary Material, further inquiries can be directed to the corresponding authors.

## Supplementary Materials

The following supporting information can be downloaded at https://www.mdpi.com/article/10.3390/polym17233212/s1, Figure S1: Fourier transform infrared spectra of halloysite and halloysite_CMC composite; Figure S2: Release of trypsin from nanotubes suspended in water (pH 5.8) after 24 h of incubation (*n* = 3); Figure S3: Averaged normalized darkfield reflectance spectrum of halloysite in the range of 420–950 nm.

## Abbreviations

The following abbreviations are used in this manuscript:

*E. coli* OP 50-1.	*Escherichia coli strain* OP 50-1
*S. aureus* 6583	*Staphylococcus aureus* 6583
CMC	carboxymethylcellulose
FTIR-ATR	Fourier transform infrared spectroscopy Attenuated total reflection
HNT	halloysite nanotubes
Lys	lysozyme
Trp	trypsin
