# Supplementary material for: Immobilization of Trypsin and Lysozyme in Halloysite Nanotubes for Producing Chitosan Coatings with Antibacterial Properties
Source: Polymers (Basel). 2025 Dec 2;17(23):3212. doi: 10.3390/polym17233212 (PMC12694494; doi:10.3390/polym17233212)
Supplement: Supplementary file 1 [file polymers-17-03212-s001.zip › polymers-3947336-supplementary.pdf]

Supporting Information

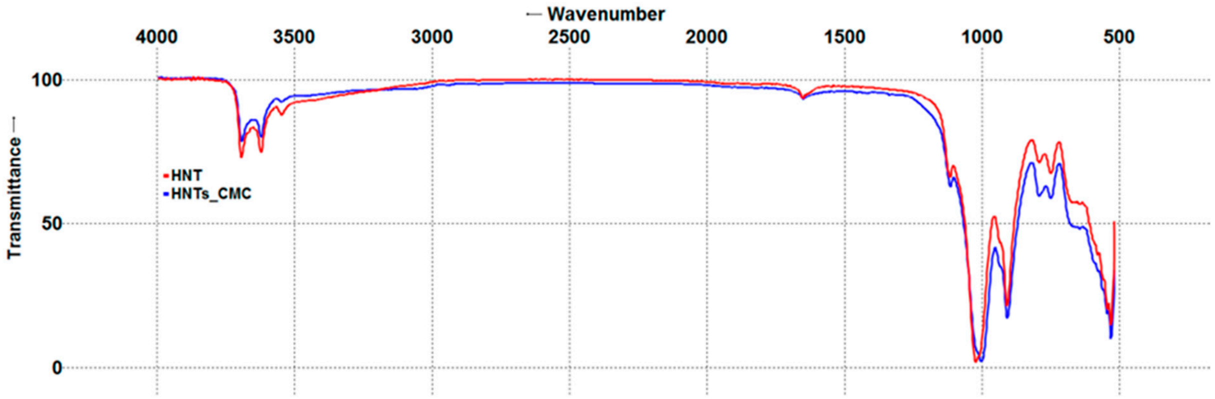

Supplementary Figure S1. Fourier transform infrared spectra of halloysite and halloysite_CMC composite

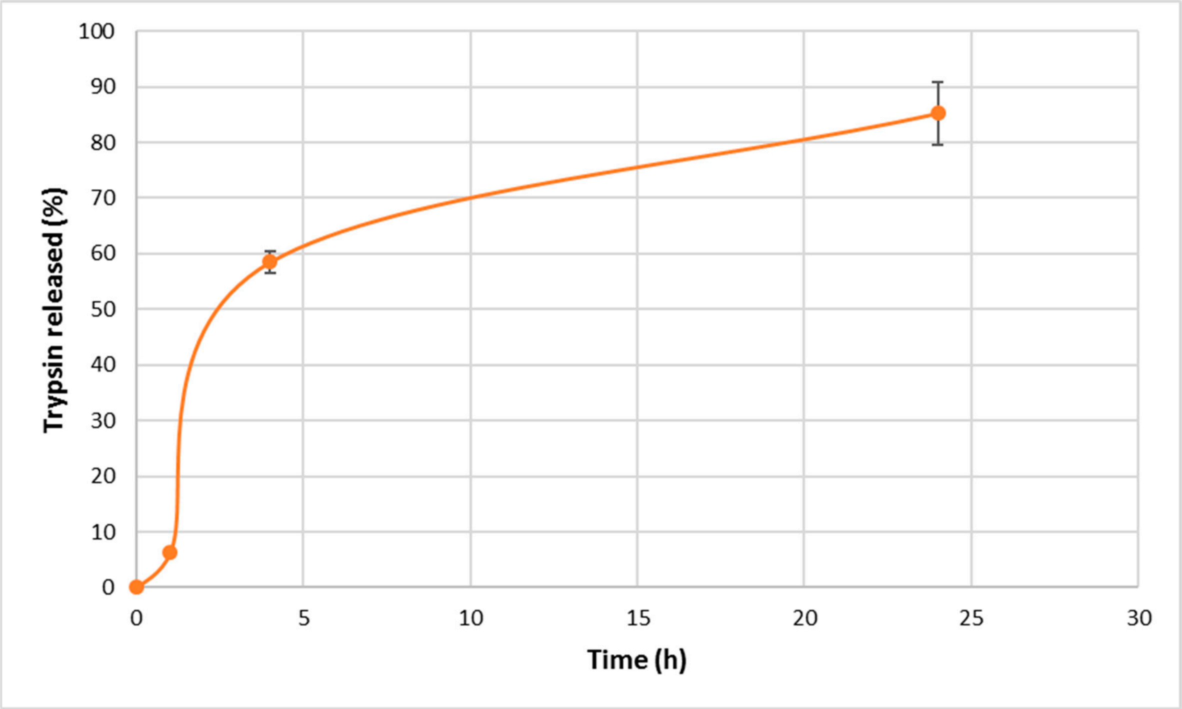

Supplementary Figure S2. Release of trypsin from nanotubes suspended in water (pH 5.8) after 24 h of incubation (n = 3).

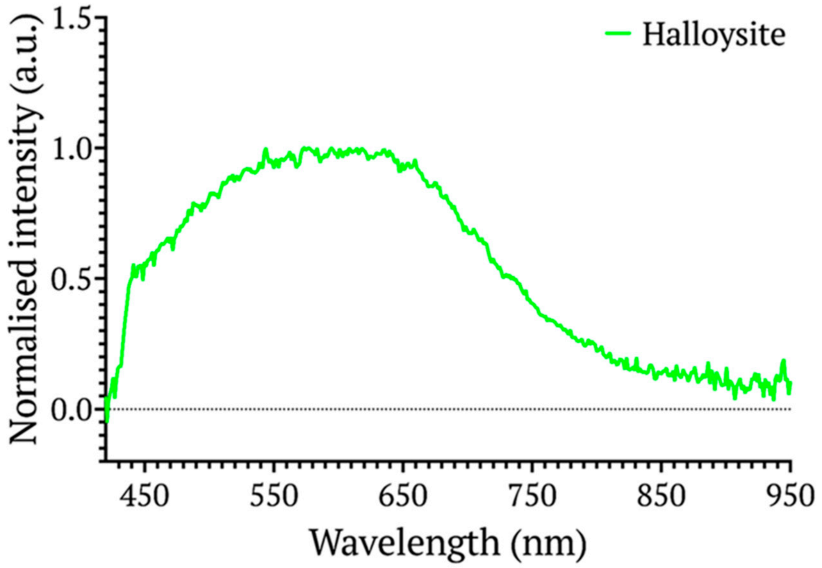

Supplementary Figure S3. Averaged normalized darkfield reflectance spectrum of halloysite in the range of 420-950 nm
